# The role of single-cell sequencing in studying tumour evolution

**DOI:** 10.12703/r/10-49

**Published:** 2021-05-26

**Authors:** Maximilian Mossner, Ann-Marie C Baker, Trevor A Graham

**Affiliations:** 1 Evolution and Cancer Laboratory, Centre for Genomics and Computational Biology, Barts Cancer Institute, Barts and the London School of Medicine and Dentistry, Queen Mary University of London, Charterhouse Square, London, EC1M 6BQ, UK

**Keywords:** single cell sequencing, tumour evolution, scRNA-Seq, scDNA-Seq

## Abstract

Tumour evolution is a complex interplay between the acquisition of somatic (epi)genomic changes in tumour cells and the phenotypic consequences they cause, all in the context of a changing microenvironment. Single-cell sequencing offers a window into this dynamic process at the ultimate resolution of individual cells. In this review, we discuss the transformative insight offered by single-cell sequencing technologies for understanding tumour evolution.

## Introduction

Intra-tumour heterogeneity (ITH) and evolution are deeply intertwined. Heterogeneity is “fuel to the fire” of evolution – the population of cells in a tumour cannot adapt to a new selection pressure if there is no variation in the population for natural selection to operate upon. And because new (epi)genetic mutations are acquired each time a tumour cell divides, the process of tumour growth (and so tumour evolution) inevitably generates heterogeneity within a tumour^1^.

For many decades, the prevalent opinion was that tumours generally follow a strictly “linear” evolutionary trajectory whereby the sequential addition of “driver” genomic aberrations was associated with a clonal expansion leading to replacement of the complete tumour population. In this paradigm, tumour progression followed with the accumulation of driver alterations^2^. However, multi-region sequencing of bulk tumour tissues has now shown that ITH, in particular the frequent coexistence of subclones with different driver alterations, is common in all tumour types, prominently including lung^3,4^, hematologic^5,6^, colon^7,8^, breast^9,10^ and brain^11,12^ neoplasia.

A mechanistic understanding of the process of carcinogenesis requires understanding the drivers and dynamics of tumour evolution^13^. Consequently, predicting and indeed modulating the response of a tumour to treatment^14^, or whether a premalignant lesion will become tumourous in the future^15,16^, both require predicting tumour evolution^16^. Indeed, recent work suggests that evolutionary trajectories are recurrent across tumours, and biomarkers that identify these trajectories could enable personalised tumour medicine^17–19^.

ITH is a read-out of tumour evolutionary dynamics^20^ and consequently measurement of ITH has prognostic relevance: pan-tumour analyses of whole exome sequencing data from The Cancer Genome Atlas revealed that tumours harbouring between two and four detectable clones have poorer prognoses than those containing fewer than two or more than four clones^21^, and clonal diversity of copy number alterations predicts survival in lung tumours^22^. Treatment resistance, tumour relapse and development of metastases can coincide with the expansion of “new” clones that harbour specific driver genomic alterations, conferring phenotypic advantages in the changing microenvironment^5,6,23^. In this context, one should be careful not to mistake passenger mutations arising through neutral evolution as contributors to evolutionary adaptation since somatic mutations accumulate randomly throughout cell divisions and many have no apparent effect on the fitness of a (sub)clone^8^. One considerable caveat in the assessment of ITH is the fact that generally we can sample only a small fraction of the entire tumour, thus limiting our ability to detect minor subclones. Extrapolating conclusions to the bulk tumour therefore should be done with caution. Translating measurements of tumour evolution, such as quantification of ITH, will require tumour sampling to be standardised (for example, number, position and size of biopsies) and tumour cellularity to be considered as these factors can confound interpretation of molecular composition^24^.

However, recent data indicates that even in tumour populations with largely identical genomes, a high degree of cell *plasticity* – extensive phenotypic cell-to-cell variability – is observed^25–28^, which can be caused by, among other factors, heterogeneity at the transcriptomic or epigenomic level or both. From an evolutionary perspective, this is a highly important notion as it is tumour cell phenotypes and not genotypes that are undergoing selection.

**Figure 1.**
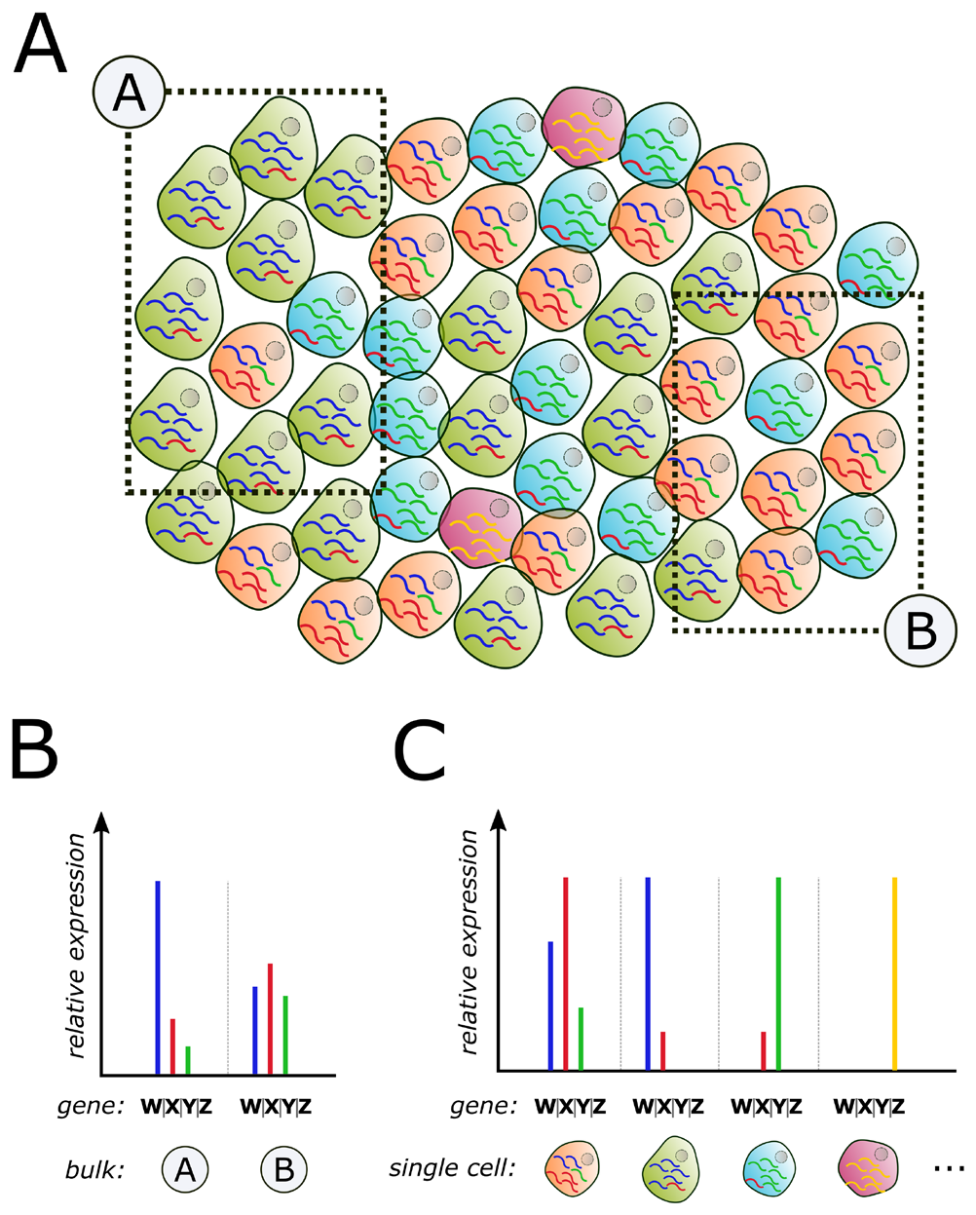
Dissecting molecular profiles of heterogeneous tumour cell populations by using bulk and single-cell methodology. (**A**) Intra-tumour heterogeneity usually manifests in the form of mixed cell populations with diverse molecular traits. As illustrated in this example, many tumours frequently show the coexistence of cell clones with different transcriptomic makeups and strongly varying population sizes. Although analysis of multicellular bulk pieces “A” and “B” reveals differences in the RNA expression profiles, these could be (inaccurately) attributed as intrinsic, tumour cell–specific changes (**B**). Only by using single-cell next-generation sequencing analysis, transcriptome profiles can be directly associated with specific cell types (such as tumour and various types of stromal cells) and allow the cell type complexity within heterogeneous tumour lesions to be enumerated (**C**). Importantly, only single-cell screening allows sensitive *de novo* identification of rare cell subpopulations that otherwise would be missed entirely or lie below the level of detection when bulk tissue analysis is used.

Next-generation sequencing (NGS) techniques have contributed tremendously to our knowledge of the molecular architectures of tumours. However, the majority of data has been generated using DNA or RNA derived from “bulk” tumour tissue (Figure 1A), almost inevitably consisting of complex mixtures of tumour cells and stromal cells, such as fibroblasts, macrophages and lymphocytes, which confound the analysis. Bioinformatics tools have been created to deconvolute the individual cell types from bulk RNA sequencing (RNA-Seq)^29–32^ but are complicated by the necessity for prior knowledge of transcriptome patterns that uniquely determine each cell type so that the signal from these cells can be isolated in the downstream analysis. However, since there is a dynamic interplay between tumour cells and their microenvironment, it is reasonable to expect that RNA expression of the same cell “type” can show inter-tumour and intra-tumour variability in gene expression^33^ (Figure 1A, B) and furthermore that this variation is an important determinant of tumour biology. Thus, bulk analysis risks missing important tumour biology.

The recent evolution of powerful single-cell sequencing techniques provides a unique opportunity to directly overcome these limitations, allowing highly unbiased assessments of tumour heterogeneity – both subclonal heterogeneity of tumour cells and the cell types present in the stroma – with an unprecedented level of sensitivity (Figure 1A, C). In the following sections, we provide an overview of common methods and recent innovations achieved through the application of single-cell analysis.

## Single-cell sequencing in tumours

### Uni-modal techniques

One of the first single-cell sequencing applications was a single-cell RNA-Seq (scRNA-Seq) protocol established in 2009 by Tang *et al*.^34^. This paved the way for a vast range of highly sophisticated single-cell next generation sequencing (scNGS) methods that characterise genomic, transcriptomic, epigenomic and proteomic patterns that will be explained in more detail below. To provide a quick overview, a summary of common methods can be viewed in Table 1.

**Table 1.** Overview of commonly used single-cell next-generation sequencing techniques.

Type of analysis	Method	Application/Features
RNA sequencing	SMART-Seq2^35^	- Full-length mRNA sequencing
	SMART-Seq3^36^	- Full-length mRNA sequencing - UMI support
	Quartz-Seq^37^	- Full-length mRNA sequencing
	MATQ-Seq^38^	- Full-length mRNA + lncRNA sequencing
	SUPeR-Seq^39^	- Full-length mRNA + lncRNA sequencing
	Holo-Seq^40^	- Full-length mRNA + lncRNA + small RNA sequencing
	MARS-Seq2^41^	- 3-prime end mRNA sequencing - UMI support
	CEL-Seq2^42^	- 3-prime end mRNA sequencing - UMI support
	mcSCRB-Seq^43^	- 3-prime end mRNA sequencing - UMI support
	STRT-Seq^44^	- 5-prime end mRNA sequencing - UMI support
DNA sequencing	Degenerate oligonucleotide primed polymerase chain reaction^45^	++CNV / −SNV detection
	Multiple displacement amplification^46–49^	−CNV / +++SNV detection
	Ampli1^50^	++CNV / +SNV detection
	STRAND-Seq^51–53^	+++CNV detection (SNV unknown)
	“Direct library preparation”^54,55^	+++CNV / ++SNV detection
Epigenomic sequencing	Bisulfite sequencing^56,57^	- CpG DNA methylation analysis
	Drop-ChIP-Seq^58^	- Chromatin immune precipitation analysis for histone modifications
	scATAC-Seq^59,60^	- Chromatin accessibility analysis
	CUT&Tag^61^	- Targeted chromatin-protein association profiling

Abbreviations: CNV, copy number variant; lncRNA, long non-coding RNA; mRNA, messenger RNA; SNV, single-nucleotide variant; UMI, unique molecular index. Suitability of DNA sequencing applications is represented on a scale from (−) to (+++). Although a range of individual single-cell RNA sequencing (scRNA-Seq) protocols show overlapping characteristic features, the final choice of method depends primarily on the requirement for quantitative analysis (for example, number of cells) versus qualitative analysis (for example, full-length assessment for splicing/fusion transcripts). As a rule of thumb, the cost of library preparation and sequencing is related to the breadth of data per cell obtained.

By far the most extensive variety of scNGS protocols in use today are scRNA-Seq approaches, which differ in aspects such as cell isolation methods, type of transcript coverage, cDNA transcription, and library amplification. Another important aspect represents the utilisation of unique molecular identifiers (UMIs), which are random DNA sequences embedded in library adapters that uniquely “tag” a cellular RNA/DNA fragment before polymerase chain reaction (PCR) amplification. By reading UMI tags after library preparation and sequencing, PCR duplicates can be easily distinguished from biological replicates, thereby generating absolute counts of the initial fragments present.

**Figure 2.**
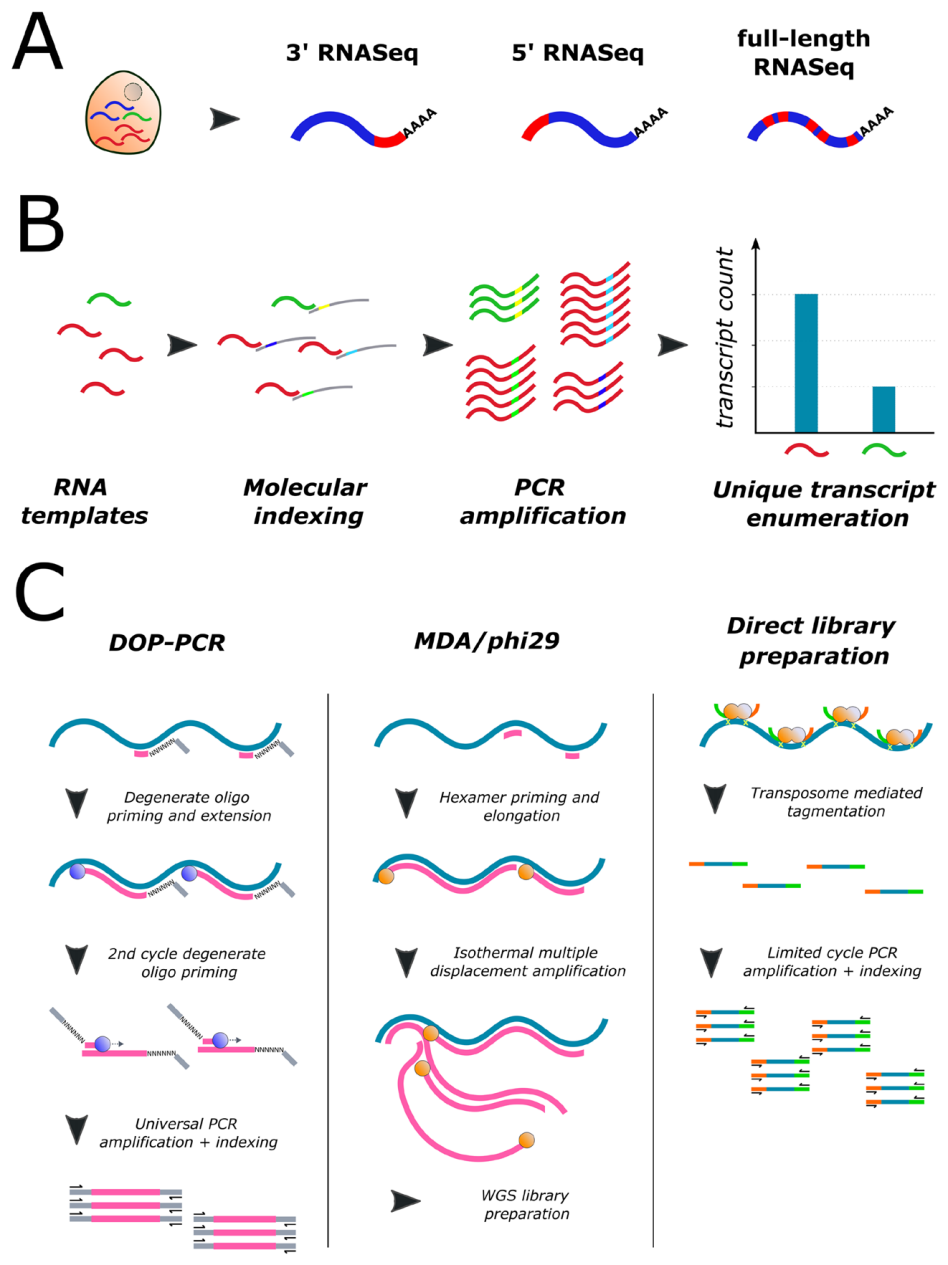
Single-cell sequencing strategies. (**A**) Single-cell RNA sequencing approaches can be split broadly into techniques that sequence either the 3′ or 5′ end of individual RNA transcripts or random parts of the full transcript molecule. (**B**) The scarcity of starting RNA templates in a single cell is a major confounding factor leading to polymerase chain reaction (PCR) amplification bias and inaccurate quantification of transcript levels. Using adapters with “unique molecular indexes” (UMIs) (random nucleotide sequences) for reverse transcription allows tagging of individual transcripts before the PCR amplification step. After sequencing and mapping of amplified libraries, the abundance of initial RNA transcripts can be estimated by counting the number of UMIs for any detected RNA transcript, thereby mitigating amplification bias. (**C**) Overview of the most common amplification techniques for assessment of genomic DNA profiles in single cells. DOP-PCR, degenerate oligonucleotide primed polymerase chain reaction; MDA, multiple displacement amplification; WGS, whole genome sequencing.

In general, scRNA-Seq protocols can be divided into full-length transcript approaches, such as SMART-Seq2^35^, Quartz-Seq^37^, MATQ-Seq^38^ or SUPeR-Seq^39^, and protocols sequencing short parts of the 3′ or 5′ end of RNA transcripts, such as MARS-Seq2^41^, CEL-Seq2^42^, mcSCRB-Seq^43^ or STRT-Seq^44^ (Figure 2A). Full-length scRNA-Seq has the advantage of identifying alternative transcript isoforms, fusion transcript events and (to a limited extent) single-nucleotide variants (SNVs) and insertion/deletion variants (INDELs) throughout entire RNA transcripts. The most commonly used full-length protocol, SMART-Seq2, has been shown to detect the largest number of transcripts per cell but exhibits higher amplification noise due to lack of UMI utilisation^62^ although this was addressed recently in the updated SMART-Seq3 protocol^36^. It also has significantly higher costs per cell unless the transposase enzyme for the final library tagmentation step is produced in-house^63^. Whereas the majority of scRNA-Seq protocols use oligo-dT priming of poly-A mRNA species, MATQ-Seq^38^, SUPeR-Seq^39^ and Holo-Seq^40^ provide total RNA expression profiles, including long non-coding RNA and circular RNA species, that are increasingly recognised as playing a key role in tumour pathogenesis. To date, Holo-Seq^40^ is the only method capable of capturing expression of small RNAs (for example, microRNAs or enhancer RNAs) in scRNA-Seq. Although these full-length methods provide exceptional insight into the complexity of single-cell transcriptomes, they inevitably require several-fold-higher sequencing depth compared with their 3′/5′ biased counterparts.

In comparison with full-length protocols, 3′ and 5′ assays benefit from the (optional but straightforward) inclusion of UMIs enabling more accurate transcript quantification (Figure 2B). In addition, the possibility to barcode RNA of individual cells means that these methods allow early multiplexing of cell samples, thereby increasing the library preparation throughput by magnitudes and reducing costs. While these protocols are predominantly carried out in various plate formats, emulsion droplet-based techniques that are able to encapsulate single cells into oil droplets for separate processing (for example, the 10x Chromium platform^64^, inDrop^65^ and Drop-Seq^66^) have recently emerged. These technologies have an even higher throughput with combined capturing and library preparation of tens of thousands of cells per sample and highly reduced library processing costs per cell. However, the drawbacks of such approaches are the high upfront infrastructure costs and the inability to map generated cell profiles to preanalytical assessments, such as fluorescence-activated cell sorting (FACS)-based epitope profiling or cellular morphology.

Single-cell approaches for genomic DNA profiling (scDNA-Seq) have experienced similar advances in the development of increasingly sensitive methods (Figure 2C). A single cell contains a minute amount of DNA (typically two copies of each allele as opposed to multiple mRNA copies of each gene) and therefore traditional scDNA-Seq protocols have required either the whole genome or loci of interest to be preamplified prior to library preparation. Generally, for whole genome amplification (WGA), isothermal multiple displacement amplification (MDA) and variations thereof have been the methods of choice because of their wide breadth of genome coverage^46,47^. However, MDA is prone to allelic imbalance/dropouts and GC amplification bias when only a single diploid genome is used as the starting template, leading to relatively high error rates in SNV/INDEL calling and less precise detection of copy number variants (CNVs)^48^. Subsequent methods used FACS to isolate cells in the G_2_/M phase of the cell cycle to increase the starting amount of DNA for WGA, which significantly reduced error rates^67^. Further optimisations of MDA and library chemistry increased SNV/INDEL detection rates to about 90%^49^ but did not improve the poor detection of CNVs. An alternative method of single-cell WGA is degenerate oligonucleotide primed PCR (DOP-PCR) and this results in high coverage uniformity and therefore very accurate CNV detection but highly limited SNV detection because of low breadth of genome coverage, usually around 10%^45^. The recent development of the *Ampli1* technology (Menarini Silicon Biosystems, Huntingdon Valley, PA, USA) improves on CNV detection and allelic dropout rates by implementing an optimised strategy using enzymatic digestion, adapter ligation followed by single-primer amplification^50^. A further alternative method called STRAND-Seq^51^, and variations thereof developed by van den Bos *et al*.^52^ and Bakker *et al*.^53^, includes a preamplification-free approach, which leads to more precise CNV karyotyping and even allows delineation of the parental origin of alleles in single cells. Generally, the choice of WGA method has to be directed by the type of genomic data that is intended to be interrogated.

An elegant approach to circumvent the problems associated with WGA has been developed by Zahn *et al*. and uses transposome tagmentation directly on single-cell genomic DNA^54^. This technique, named “direct library preparation”, uses a one-pot reaction for DNA fragmentation and adapter ligation, omits preamplification before library construction and thereby provides a wide breadth of genome coverage that allows highly accurate CNV detection even with low coverage sequencing (<0.1×). In this approach, much like in the above-mentioned STRAND-Seq method^52,53^, the lack of preamplification allows researchers to easily identify and remove PCR duplicates because of the unique fragmentation pattern of a single-cell genome, resulting in vastly improved noise reduction. Intriguingly, genome allelic balance remains largely preserved and enables sensitive SNV/INDEL calling by combining single-cell genomes *in silico* to create a “synthetic bulk”; for example, single cells are assigned to a subclonal population by using their CNV profiles derived from the same scDNA-Seq dataset. More recently, the method has been scaled to allow processing of tens of thousands of cells with compatibility to commercially available single-cell processing solutions^55^.

Although the above-mentioned techniques allow deep insight into ITH at single-cell resolution, spatial information is lost because in the generation of single-cell suspensions for library preparation the tissue architecture is destroyed. To address this problem, several groups developed *in situ* analysis technologies^68–70^ that have single-cell – or near cellular – resolution. For example, our group recently validated BaseScope for robust detection of mutation-specific RNA as a means to measure ITH in a spatial context^71^. The higher-throughput “slide-seq” method interrogates global RNA expression profiles with single-cell resolution in virtually any type of sectioned tissue^70^. To achieve this, the authors engineered arrays of 10-μm, uniquely barcoded beads with known positions that capture mRNA species upon transfer from tissue sections via oligo-dT priming, enabling high-resolution spatial transcriptome mapping of tens of thousands of single cells per section to be achieved. In theory, genetic information (CNVs and potentially SNVs) extracted from the RNA data can be used to spatially map genotypes concurrently with the RNA-based phenotypic characterisation. Of note, by implementing an innovative *in situ* RNA-Seq approach, STARmap expands on the possibilities of slide-seq and enables even three-dimensional spatial single-cell transcriptomic profile resolution^69^.

Recent advances in single-cell processing have also allowed tumour epigenetics to be investigated on a larger scale and at cellular resolution. Widely used methods include single-cell bisulfite sequencing for measuring DNA CpG methylation^56,57^, droplet-based single-cell chromatin immunoprecipitation (drop-ChIP) for assessment of histone modifications^58^, single-cell transposase-accessible chromatin sequencing (scATAC-Seq) for analysing chromatin accessibility^59,60^ and cleavage under targets and tagmentation (CUT&Tag) for measuring custom chromatin protein distribution^61^. Although they still provide, on a per-cell level, only a small fraction of the epigenetic information obtained from bulk sample measurements, these techniques represent essential tools for connecting phenotype and genotype features of tumour evolution on a cellular level.

Analysis and interpretation of scNGS datasets must be performed with appreciation of certain caveats. For example, it is important to note that tissue dissociation protocols for the generation of single-cell suspensions can be quite harsh, causing fragile states of single cells and RNA species in particular and this may bias detection to “hardy” cells. Moreover, owing to the scarcity of template material in a single cell and hence the necessity for strong signal amplification, stochastic dropout of biological features is inevitable. As such, quality control of the data and selection of adequate computational analysis strategies are of utmost importance to ensure that the results reflect true biological heterogeneity between and within samples and not simply variable quality of input material or batch processing artefacts. Methods to robustly handle these intrinsic sources of noise are essential: Excellent reviews of scNGS computational analysis pipelines can be found elsewhere^72–76^.

### Multi-modal techniques

Mapping multiple biological features within the same cell is an important step towards understanding the molecular interdependencies that lead to phenotypic diversity within tumours. As such, there is currently much interest in combining multiple uni-modal single-cell techniques to develop multi-modal scNGS approaches. For example, TARGET-Seq combines an improved SMART-Seq2 scRNA-Seq protocol with targeted SNV/INDEL genotyping by using custom primers amplifying loci from both RNA and DNA in the same reaction, thereby dramatically improving the high allelic dropout rate usually observed with mutation calling using (full-length) scRNA-Seq data only^77^. Similar approaches for successful mapping of transcriptome and targeted genotype profiles of single cells have been reported by two other groups^78,79^ using modifications of 3′ scRNA-Seq protocols. The use of 3′ RNA-Seq, in comparison with TARGET-Seq, as a genotyping source might be a limiting factor for the genotyping success rate as mutations in the 5′ region as well as in marginally expressed genes may not be detected. Though significantly expanding on our abilities to unify genotypic and transcriptomic cell profiles, the limitation of the above methodologies is that prior knowledge of the mutations to be interrogated is required. Combined genome and transcriptome sequencing (“G&T-Seq”) has been the first method to provide a more unbiased screening solution and allows the assessment of complex genomic profiles in parallel with transcriptomic profiles^80,81^. With biotinylated oligo-dT capture probes, RNA moieties of a single cell can be physically separated from the DNA and both can be subjected independently to the aforementioned scNGS protocols.

The REAP-Seq and CITE-Seq protocols combine scRNA-Seq and cell-surface proteomics by labelling single cells with antibody–oligonucleotide conjugates before extracting RNA^82,83^. The oligonucleotides of these conjugates carry epitope-specific barcodes and poly-A tails and therefore can be conveniently quantified by integrating them in common scRNA-Seq library protocols. In a similar approach, the RAID-Seq method employs scRNA-Seq of fixed single cells and simultaneously allows intracellular (phospho)proteins to be quantified^84^. These approaches are compatible with high-throughput microfluidics platforms, such as that of 10x Genomics, and can be highly multiplexed to simultaneously assess dozens of protein epitopes and the corresponding transcriptomic profile of a single cell.

## Insights from single-cell next-generation sequencing into tumour evolution

With a highly effective arsenal of single-cell methods at hand today, recent studies have revealed exciting novel insights into tumour heterogeneity and evolutionary principles. For example, Gao *et al*. investigated patterns of aneuploidy in triple-negative breast tumour (TNBC) by generating genome-wide CNV profiles of 1,000 single cells from 12 patients^85^. Interestingly, the investigators’ analysis revealed highly stable karyotypes across individual tumour clones and only rare events of “metastable” tumour cells showed additional single aberrations, presumably denoting evolutionary dead ends. The data was consistent with a mathematical model whereby CNVs were acquired in a “punctuated burst” early in TNBC tumourigenesis rather than through gradual acquisition. Notably, though, the model did not describe the effects of selection – which could conceivably lead to a clonal expansion and the clonality of CNVs observed in the data.

Another study characterised oestrogen receptor–positive breast tumour via scRNA-Seq in combination with cellular barcoding analysis of bulk tumour cells (the ClonTracer system)^86^, revealing that resistance to anti-oestrogen therapy occurs mainly through selection of pre-existing clones^87^. Notably, resistance development appears to be further promoted by increased activity of the KDM5 histone demethylase family, which is frequently observed in patients, mainly by epigenetically induced enhanced transcriptomic heterogeneity, and can be reversed by KDM5 inhibition. Intriguingly, cellular barcoding in conjunction with scRNA-Seq data also indicated that, in contrast to anti-oestrogen treatment, eventual acquisition of resistance to KDM5 inhibition is attributed to epigenetic and not genetic changes, highlighting different paths of evolutionary adaptation to therapy.

Subjecting TNBC single-cell to scDNA-Seq, followed by longitudinal analysis in TNBC tumours before, during, and after chemotherapy, also revealed the outgrowth of rare pre-existing clones upon chemoresistance development^88^, underlining the clinical relevance of measuring ITH. Intriguingly, the parallel scRNA-Seq analysis on a separate set of single cells from the same tissues uncovered that resistance-associated transcriptional profiles partly pre-existed and were further reshaped during therapy to fully establish the transcriptional signature of phenotypic resistance^88^ via upregulation of receptor tyrosine kinase, epithelial-mesenchymal transition and immune-associated expression pathways^25^.

H3K27M-mutant glioma represents a highly fatal tumour subtype that occurs during early childhood and is assumed to be linked to the activation of proliferative brain development programs. By using scRNA-Seq, Filbin *et al*. were able, for the first time, to delineate the phenotypic composition of these tumours at cellular resolution, revealing that they are composed mainly of a stem-like oligodendrocyte precursor subset with highly proliferative and tumour-propagating characteristics but that differentiated cells represent only a minority of the tumour population^89^. Notably, complementing their primary tumour analysis with patient-derived xenografts indicated that platelet-derived growth factor receptor alpha (PDGFRA) and BMI1 are involved in maintaining the stem-like phenotype and therefore represent potential therapeutic targets in this inoperable and aggressive disease.

Although scRNA-Seq profiling provides detailed insight into cell-specific transcriptional states, some tumour types such as acute myeloid leukaemia (AML) can to a certain extent show a variety of differentiation phenotypes similar to that observed in healthy tissue homeostasis, which makes it necessary to accurately determine cell genotypes in parallel. As described earlier, van Galen *et al*. performed simultaneous scRNA-Seq and targeted sequencing of patient-specific mutations (present in RNA transcripts) from the very same cell^79^. By using a machine learning classifier, they revealed that AML single-cell transcriptomes indeed closely resembled up to six normal myeloid differentiation states and their compositions were highly variable between patients and associated with genetic background and clinical outcome. Furthermore, their approach allowed unbiased assessment of AML stem-like cells, which revealed a strong upregulation of stress response, redox signalling and self-renewal programs compared with normal hematopoietic stem cells and identified CD36 and CD74 as novel putative leukaemia stem cell markers for potential therapeutic exploitation.

In an effort to unravel the complex relationship between tumour and stromal cells, recent studies from the Teichman lab using scRNA-Seq analyses in human- and mouse-derived melanomas revealed the requirement of a delicate balance of the immune complement system for maintaining tumour growth^90^. The group’s “CellPhoneDB” tool uses multicellular scRNA-Seq data to identify cell–cell communication networks by identifying receptor–ligand pairings that underlie biological multicellular interactions^91^.

Collectively, although the findings described here are only a small representation of recent achievements made possible by single-cell investigations, they highlight the immediate clinical relevance of scNGS and the potential to transform our understanding of tumour ITH and mechanisms of clonal evolution.

## Conclusions

Single-cell sequencing techniques have proven to be powerful tools to dissect the heterogeneity of tumour cells. Investigating the genomic, transcriptomic and epigenomic features of individual tumour and stromal cells within a tumour, and their interactions, already revealed important biological mechanisms underlying the propagation and maintenance of tumour development. The ongoing refinement of these methods and vast increase in throughput will soon allow even deeper and more precise characterisation of spatial and temporal tumour evolution, microenvironmental interactions and immune crosstalk. The exciting new possibilities offered by single-cell technologies are likely to translate to new prognostic tools and personalised treatment strategies in the near future, extending the survival of tumour patients and improving clinical management.

## Abbreviations

AML, acute myeloid leukaemia; CNV, copy number variant; FACS, fluorescence-activated cell sorting; INDEL, insertion/deletion variant; ITH, intra-tumour heterogeneity; MDA, multiple displacement amplification; NGS, next generation sequencing; PCR, polymerase chain reaction; RNA-Seq, RNA sequencing; scDNA-Seq, single-cell DNA sequencing; scNGS, single-cell next-generation sequencing; scRNA-Seq, single-cell RNA sequencing; SNV, single-nucleotide variant; TNBC, triple-negative breast cancer; UMI, unique molecular identifier; WGA, whole genome amplification
